# Non-coding RNAs: Emerging Regulators of Sorafenib Resistance in Hepatocellular Carcinoma

**DOI:** 10.3389/fonc.2019.01156

**Published:** 2019-11-05

**Authors:** Yongting Lai, Bing Feng, Mubalake Abudoureyimu, Yingru Zhi, Hao Zhou, Ting Wang, Xiaoyuan Chu, Ping Chen, Rui Wang

**Affiliations:** ^1^Department of Medical Oncology, Nanjing School of Clinical Medicine, Jinling Hospital, Southern Medical University, Nanjing, China; ^2^Department of Medical Oncology, School of Medicine, Jinling Hospital, Nanjing University, Nanjing, China; ^3^Department of Medical Oncology, Jinling Hospital, Nanjing Medical University, Nanjing, China; ^4^Department of Oncology, First People's Hospital of Yancheng, Fourth Affiliated Hospital of Nantong University, Yancheng, China

**Keywords:** hepatocellular carcinoma (HCC), sorafenib resistance, non-coding RNAs (ncRNAs), microRNAs (miRNAs), long non-coding RNAs (lncRNAs)

## Abstract

As the first oral multi-target anti-tumor drug proved for the treatment of patients with advanced liver cancer in 2007, sorafenib has changed the landscape of advanced hepatocellular carcinoma (HCC) treatment. However, drug resistance largely hinders its clinical application. Non-coding RNAs (ncRNAs), including microRNAs (miRNAs), and long non-coding (lncRNAs), have recently been demonstrated playing critical roles in a variety of cancers including HCC, while the mechanisms of ncRNAs in HCC sorafenib resistance have not been extensively characterized yet. Herein, we summarize the mechanisms of recently reported ncRNAs involved in sorafenib resistance and discuss the potential strategies for their application in the battle against HCC.

## Introduction

Liver cancer is one of the most commonly diagnosed malignancies in clinic. The annual death of liver cancer is about 782,000, ranking the 4th leading cause of cancer-related mortality worldwide in 2018, with HCC accounting for 75–85% among them (1). China is the highest incidence area of HCC, accounting for more than 50% of the world's burden (2). Despite the advances in diagnostic and therapeutic techniques, the 5-years survival rate of HCC continues to be very low. First, the disease is often diagnosed at an advanced stage, most of the patients will not be able to benefit of curative strategies (3). In addition, HCC is usually associated with liver function impairment, limiting the efficacy of chemotherapy as the drug doses must be reduced to avoid intolerable side effects in these patients (4).

The emergence of sorafenib has brought new hope to the treatment of patients with advanced liver cancer. Sorafenib is the first first-line systemic treatments proven by the U.S. FDA. The phase 3 SHARP (Sorafenib HCC Assessment Randomized Protocol) study demonstrates an overall survival (OS) benefit of sorafenib vs. placebo (10.7 vs. 7.9 months) (5–7). Similar results are reported in a phase 3 study from the Asia-Pacific (AP) region (6.5 vs. 4.2 months) (8). As an oral multi-kinase inhibitor, sorafenib exerts its functions mainly through mechanisms of anti-tumor cell proliferation and anti-angiogenesis. First, sorafenib directly inhibits tumor cell proliferation by targeting multiple kinases involved in the Ras/Raf/MEK/ERK signaling pathway, including Raf-1, B-Raf. Furthermore, sorafenib indirectly inhibits tumor cell proliferation by suppressing tumor angiogenesis via targeting c-Kit, FLT-3, VEGFR-2/3, PDGFR-β, and other tyrosine kinases, which are activated in tumor angiogenesis. Meanwhile, sorafenib could also induce cell apoptosis by targeting Mcl-1 (5, 6).

However, only 30% of HCC patients could benefit from sorafenib and the acquired resistance often happens within 6 months (6). The high incidence of sorafenib resistance has become a limiting factor in its clinical application, while the underlying mechanisms remain largely unknown. Currently, a series of factors have been proved closely correlated with sorafenib resistance, including apoptosis resistance, dysregulation of cell-cycle, drug efflux system, drug metabolism, epithelial–mesenchymal transition (EMT), cancer stem cell (CSC) generation, epigenetics, and microenvironment including angiogenesis, autophagy, hypoxia, inflammation viral activation and activation of pivotal signal pathways, such as PI3K/AKT Pathway, MAPK Pathway, TGF-β pathway et al.

**Figure 1:**
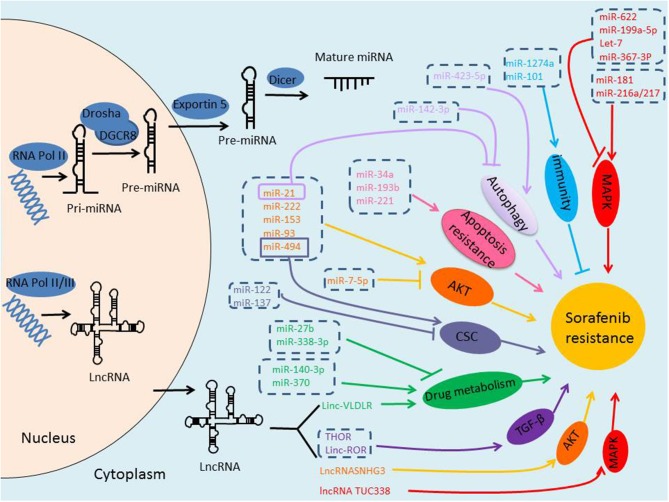
Biogenesis of ncRNAs and the role of ncRNAs in sorafenib-resistance of HCC. miRNAs are transcribed by RNA Polymerase II, and the initial product is pri-miRNA, pri-miRNA is processed into a pre-miRNA by Drosha/DGCR8 in the nucleus, and then transported to the cytoplasm via Exportin-5, pre-miRNA is further cleaved by Dicer to form mature miRNA. lncRNAs are transcribed by RNA Polymerase II or III. Either miRNAs or lncRNAs, may play critical roles in regulating HCC resistance to sorafenib through different signal pathways and mechanisms.

NcRNAs are defined as a kind of RNAs with no protein-coding potential, which account for the majority of RNAs (9). Recently, it has been increasingly recognized the important role of ncRNAs in many physiological and pathological processes. With the rapid progress in the identification of new ncRNAs, a large body of literature has indicated that ncRNAs, mainly miRNAs and lncRNAs, may play critical roles in regulating HCC resistance to sorafenib through different signal pathways and mechanisms, suggesting them as potentially novel prognostic markers, and attractive therapeutic molecules in conquering sorafenib resistance (Figure 1).

## miRNAs Involved in Sorafenib Resistance

Defined as small non-coding RNA with 20–22 nt in length, miRNAs are transcribed by RNA Polymerase II and displaying functions through binding to the 3′-untranslated regions UTR (3′-UTR) of the targeted mRNAs, thereby decreasing the expression of these specific mRNAs by either mRNA cleavage or translational repression (10). Each gene can be influenced by multiple miRNAs and one miRNA may regulate multiple target genes (11, 12).

Emerging evidence indicates that changes in miRNA expression profiles could influence the chemo- and/or radio-sensitivity of cancer cells including HCC cells (13). Mechanisms include but are not limited to PI3K/AKT signaling pathway, MAPK signaling pathway, TGFβ signaling pathway, autophagy, liver CSC generation, and so on. The related research contents are listed in Table 1.

**Table 1:** miRNAs involved in sorafenib resistance in HCC.

**microRNA**	**Effect**	**Target**	**Mechanism**	**References**
miR-7-5p	Increase	TYRO3	Inactivate PI3K/AKT	(14)
miR-222	Decrease	PTEN	Activate PI3K/AKT	(15)
miR-153	Decrease	PTEN	Activate PI3K/AKT	(16–18)
miR-93	Decrease	CDKN1A PTEN	Apoptosis resistance Activate PI3K/AKT	(19)
miR-622	Increase	KRAS	Inactivate MAPK	(20)
miR-181a	Decrease	RASSF1	Activate MAPK	(21)
miR-199a-5p	Increase	MAP4K3	Inactivate MAPK	(22)
miR-216a /217	Decrease	SMAD7 PTEN	Activate MAPK	(23)
miR-34a	Increase	Bcl-2	Promote apoptosis Activate Notch-1	(24)
miR-193b	Increase	Mcl-1	Promote apoptosis Activate HCV	(25)
miR-193a	Decrease	uPA	Promote invasion and metastasis	(26)
let-7	Increase	Bcl-xl Caspase-3	Promote apoptosis Inactivate MAPK	(27) (22)
miR-221	Decrease	—	Apoptosis resistance Inactivate caspase-3/7	(28)
miR-125a	Increase	—	Block cell-cycle	(29)
miR-486	Increase	CITRON CLDN10	Inhibit EMT	(30)
miR-21	Decrease	PTEN	Inhibit autophagy Activate PI3K/AKT	(31)
miR-423-5p	Increase	—	Promote autophagy	(32)
miR-142-3p	Increase	ATG5 ATG16L1	Inhibit autophagy	(33)
miR-122	Increase	PDK4 GALNT10 SLC7A1	Inhibit CSC formation Promote apoptosis	(34) (35) (36)
miR-494	Decrease	p27, PTEN, puma	Promote CSC formation Activate PI3K/AKT Apoptosis resistance	(37) (38)
miR-137	Increase	ANT2	Inhibit CSC formation	(39)
miR-27b	Increase	CCNG1 CYP1B1	Suppress drug metabolism	(40)
miR-338-3p	Increase	HIF-1α	Suppress drug metabolism	(41)
miR-379	Decrease	—	Facilitate drug metabolism	(42)
miR-140-3p	Increase	PXR	Suppress drug metabolism	(43)
miR-1274a	Increase	ADAM9	Increase antitumor immunity	(44)
miR-101	Increase	DUSP1	Inactivate TGF-β Increase antitumor immunity	(45)
miR-367-3p	Increase	MDM2 AR	inactivate MAPK	(46)

*“—“: unknown*.

*PI3K, phosphatidylinositol 3-kinase; AKT, protein kinase B; PTEN, phosphatase and tensin homolog; CDKN1A, cyclin-dependent kinase inhibitor 1, MAPK, mitogen-activated protein kinase; RASSF1, Ras association domain family member 1; MAP4K3, The serine/threonine kinase GCK-like kinase; SMAD7, drosophila mothers against decapentaplegic protein 7; Bcl-2, B cell lymphoma/lewkmia-2; HBV, hepatitis B virus; HCV, hepatitis C virus; Mcl-1, myeloid cell leukemia-1; caspase-3, cysteinyl aspartate specific proteinase; HCC, hepatocellular carcinoma; uPA, urokinase-type plasminogen activato;, MMP-11, matrix metalloproteinases; Zbtb7a, zinc finger and BTB domain containing 7a; EMT, epithelial-mesenchymal transition; PDK4, pyruvate dehydrogenase kinase 4; SLC7A1, arginine transporter-solute carrier family 7; GALNT10, galactosaminyltransferase-10; ATG5, autophsgy-related protein 5; ATG16L1, autophagy related protein 16 like protein 1; CSCs, cancer stem cells; PUMA, P53-upregulated-modulator-of-apoptosis; SOCS-1, suppressor of cytokine signaling-1; ANT2, adenine nucleotide translocator 2; CCNG1, Cyclin-G1; CYP1B1, cytochrome P4501B1; HIF-1α, hypoxia-inducible factor-1α; MRP2, multi drug resistant protein; PXR, pregnenolone X receptor; ADAM9, proteolytic and metalloproteinase 9; DUSP1, dual specificity phosphatase 1; TGF-β, transforming growth factor β; MRP2, multi drug resistant protein; AR, Androgen receptor*.

### miRNAs and PI3K/AKT Signaling Pathway

As a tumor survival mechanism, the activation of PI3K/AKT pathway has been highlighted in failure against sorafenib-induced cell death of HCC cells (47). On the contrary, suppression of PI3K/AKT pathway activation may resensitize HCC cells to sorafenib again (48).

miRNAs participate in the activation of PI3K/AKT signaling pathway by binding to the 3′-UTR of the targeted mRNAs, in particularly phosphatase, and tensin homolog (PTEN), which is a negative regulator in PI3K/AKT pathway, For example, miR-21, miR-222, miR-494, miR93, and miR153 are all proved inducers of sorafenib resistance in HCC cells by targeting PTEN (15, 16, 19, 31, 49).

On the other hand, miRNAs are also reported to enhance the efficacy of sorafenib by inhibiting the activity of the PI3K/AKT/mTOR signaling pathway. For instance, miR-7 is suggested to reverse sorafenib resistance in HCC by suppressing the PI3-Kinase/AKT signal transduction pathway via target TYRO3, a member of the TAM family of receptor tyrosine kinases (RTKs) (14). TAM RTK has been shown to be dysregulated in a variety of tumors, and its activation in multiple survival signaling pathways could promote tumor cell survival, drug resistance, migration, and invasion (50). Moreover, its expression level is positively correlated with AFP expression and tumor diameter (51).

### miRNAs and MAPK Signaling Pathway

MAPK pathway regulates a variety of important cellular physiological and pathological processes including cell growth, differentiation, inflammatory response, hypoxia response, and drug resistance. As in HCC, knockout of MAPK14 (p38a) increases the therapeutic efficacy of sorafenib in sorafenib resistance rat model.

Recently, several miRNAs have been proved to participate in sorafenib resistance by altering MAPK signaling pathway activation. For instance, miR-622 is identified as a reversor in sorafenib resistant HCC cells by targeting KRAS, one isoform type of RAS, known as the upstream activator of RAF/MAPK pathways (20). Overexpression of miRNA-622 could significantly attenuate the activation of ERK and AKT in HCC cells and reverse the resistance of sorafenib. Besides, miR-181a evokes sorafenib resistance through direct targeting of RASSF1, which plays a negative regulatory role in MAPK activation (21). The effects of sorafenib is weakened in HCC cells after RASSF1 knockdown, endowing HCC cells with a survival advantage (21, 46). In addition, miR-199a-5p could cooperate with let-7c and increase sorafenib sensitivity of HCC cells by directly targeting MAP4K3 (MEKKK3, MAPKKKK3), which then regulates MAP4K3 expression, together playing an important role in inhibiting migration, invasion and metastasis of HCC cells (22).

### miRNAs and Apoptosis Resistance and Dysregulation of Cell Cycle Regulation

Apoptosis is a basic cellular biological phenomenon, and regarded as “self-conscious suicide behavior” of the cell. Multiple anti-apoptotic and pro-apoptotic factors are involved in this process, such as the Bcl-2 family, the caspase family, oncogene c-myc, and tumor suppressor gene P53 (52). Activation of anti-apoptotic proteins and/or suppression of pro-apoptotic proteins could lead to apoptosis resistance in cancer cells.

Several miRNAs have been validated to elevate the efficacy of sorafenib by targeting the anti-apoptotic mRNAs, such as Bcl-2, Bcl-XL, Mcl-1. For example, miR-193b augments the efficacy of sorafenib on hepatoma cells by targeting Mcl-1 (25). Let-7 miRNA potentiates the effects of sorafenib through direct targeting of Bcl-XL, an anti-apoptotic factor which belongs to the Bcl-2 family (27). On the other hand, some miRNAs are reported to facilitate HCC resistance to sorafenib by suppress activation of pro-apoptosis proteins. For instance, miR-221 over-expression could antagonize the anti-tumor effect of sorafenib on HCC through inhibiting the activation of caspase-3/7 mediated apoptosis both *in vitro* and *in vivo* (28). Moreover, miR-122 overexpression could reduce the transport of arginine and decrease NO synthesis by targeting the arginine transporter-solute carrier family 7 (SLC7A1), thereby promoting apoptosis and enhancing the effect of sorafenib (36).

The dysregulation of cell-cycle is one of the most important features of cancer cells and closely related to the development of cancer. Sorafenib arrests cell cycle by inhibiting the regulatory factors, such as p21, p27, and cyclin D1. It is suggested that inhibition of miR-125a could endow HCC cell proliferation by activating sirtuin-7, a NAD(+)-dependent deacetylase counteracting the anti-proliferative activity and sorafenib function (29). MiR-125a also shows an extra ability to inhibit angiogenesis and cell migration. Moreover, miR-486 is reported to exert positive regulation on the effect of sorafenib in hepatoma cells by targeting CITRON (30), which plays an important role in inhibiting the growth characteristics of HCC in late cytokinesis (53).

### miRNAs and Autophagy

Autophagy is a cellular degradation pathway, playing a crucial role in maintaining cellular homeostasis (54). A number of studies have indicated that autophagy could be commonly activated during chemotherapy. Recent studies have found the significant activation of autophagy in sorafenib-treated hepatoma cells in a dose-dependent pattern and the subsequent alteration of sorafenib sensitivity of HCC cells (55). However, autophagy acts as a double-edged sword in cancer (56), as well as in regulating the efficacy of sorafenib (57). On one hand, sorafenib-induced autophagy acts as a protective mechanism that attenuates the sensitivity of liver cancer cells to sorafenib (55). On the other hand, autophagy is considered to be the Type II programmed cell death (PCD), which contributes to cell apoptosis (32).

More and more miRNAs are reported to be involved in the regulation of autophagy and sorafenib sensitivity (58). For example, miR-21 up-regulation contributes to sorafenib resistance by inhibiting autophagy. The expression of miR-21 increases after HCC cells' exposure to sorafenib, coincided with upregulation of autophagy-related protein (ATG)-6 and−8. On the contrary, sorafenib-resistant HCC cells could overcome the drug-resistance by autophagy promotion via blocking miR-21 expression (31). Sorafenib treatment elevates the level of miR-423-5p expression, which enhances the anti-tumor effect of sorafenib by promoting autophagy (32).

Considering the cellular protective role of autophagy, it is not surprising that inhibition of autophagy may enhance the anti-tumor effect of sorafenib on HCC cells. For instance, the expression of miR-142-3p significantly decreases in HCC cells after exposure to sorafenib, while miR-142-3p overexpression results in a remarkable weakening in the expression of ATG5 and autophagy-related 16-like 1 (ATG16L1) and re-sensitive HCC cells to sorafenib (33). Taken together, miR-21 contributes to HCC resistance to sorafenib by inhibiting autophagy. MiR-423-5p and miR-142-3p potentiate the effect of sorafenib by modulating autophagy.

### miRNAs and CSCs

CSCs, also called tumor initiating cells, are a small subgroup of cells maintaining the self-renewal and differentiating characteristics (59) and existed in various types of cancers including HCC (60). The poor sensitivity of CSCs to chemo- and radio-therapies is an importance reason for the dim prognosis of HCC patients. It has been proved that eliminating stem-like cells in HCC can re-sensitive sorafenib-resistant cells to sorafenib (61). HCC stem cells bear multiple surface markers, including epithelial cell adhesion molecule (EpCAM), CD24, CD90, and CD133 (62–65). Various signaling pathways including JAK/STAT3, TGF-β/SMAD, Wnt, Notch, and β-catenin have been reported to regulate the activation of CSCs (61–63, 66).

Many studies show that CSCs become enriched following sorafenib treatment, suggesting that sorafenib may induce HCC cells with CSC properties. For instance, CSCs are enriched in sorafenib-resistant HCC cells and further enriched by insulin-like growth factor (IGF) and fibroblast growth factor (FGF) signaling cascades vs. sorafenib sensitive HCC (67).

Recent years, the role of miRNAs in sorafenib sensitivity regulation of HCC cells by manipulating CSC generation has been discovered. It is reported that miR-122 could suppress CD133 (+) cells stemness characteristics and overcome resistance to sorafenib by regulating glycolysis which could maintain CSCs characteristics (34). In another study, miR-122 packaged in exosome of adipose tissue derives mesenchymal stem cells (MSCs), which elevates sensitivity of sorafenib by down-regulating the expression of IGF1R, cyclin G1 (CCNG1), and ADAM10 both *in vitro* and *in vivo* (35). MiR-494 is reported to induce stem cell-like characteristics and weaken sensitive efficacy of HCC cells by targeting p27, PTEN, and p53-upregulated-modulator-of-apoptosis (PUMA) (38). The level of miR-494 is up-regulated in CSCs and a positive correlation between miR-494 and CSC markers expression, such as PROM1/CD133 and EPCAM, is detected. Moreover, miR-494 overexpression could also increase core stemness genes transporter levels including PROM1, OCT4, SOX2, and ABCG2.

In addition, let-7a and let-7b conduce to survival signaling by IL-6 in CSCs. IL-6 has anti-apoptotic effect during hepatocytes transformation and is up-regulated in liver CSCs, which may serve as an explanation for why liver CSCs are resistant to sorafenib (68). Furthermore, miR-137 up-regulation overcomes sorafenib resistance by degrading adenine nucleotide translocator 2 (ANT2) in HCC (39). ANT2 has been verified to promote sorafenib resistance and endow HCC cells with CSC phenotypes, as well as the metastasis-associated characteristics. Besides, ANT2 could also promote the expression of NURR1, DLX2, and ADRB2, and inhibit the expression of RASSF1, which weakens the CSC characteristics and migration and invasion abilities of HCC cells (69).

### miRNAs and Viral Activation

Approximately 80% of HCC cases are caused by chronic hepatitis B virus (HBV) and hepatitis C virus (HCV) infections which are common worldwide (70). It leads to not only liver function impairment but also the progression of HCC, such as hepatitis, fibrosis, and cirrhosis, finally inducing the occurrence of HCC. Moreover, HBV could also promote autophagy (71), which may partially explain sofarenib resistance of HCC.

HBV-positive HCC patients show a low survival rate according to a phase III RCT from Asia-Pacific region. Interestingly, patients with HCV infection exhibite a better prognosis median overall survival and time to progression compared with those without HCV infection according to the SHARP trial (70).

Research on the relationship between viral activation and the efficacy of sorafenib has been carried successively these years. For instance, the abundance of miR-122 is significantly inhibited in HBV-associated HCC, facilitating HBV replication and persistence. On the contrary, up-regulation of miR-122 aids HCC cells in overcoming resistance to sorafenib by targeting polypeptide N-acetyl-galactosaminyltransferase-10 (GALNT10) (36), which could induce HCC cells proliferation and apoptosis resistance in a glycosyltransferase-dependent manner.

It is also proved that the expression of miR-193b is increased in HBV/HCV-infected cells. Moreover, up-regulation of miR-193b sensitizes HBV/HCV-associated HCC cells to sorafenib mainly through targeting the anti-apoptotic protein Mcl-1 (25, 72).

### Other Mechanisms

miRNAs are also involved in other processes to regulate the response to sorafenib of HCC. For example, miR-27b can enhance sorafenib efficacy through manipulation on both the p53 pathway via miR-27b-CCNG1-p53 mechanism and drug detoxification via CYP1B1 (40). CCNG1 negatively regulates ATM-dependent p53 activation and leads to p53 degradation by recruitment of beta-subunit phosphatase 2 (PP2A) dephosphorylation of MDM2 in DNA damage. CYP1B1 is a major P450 enzyme that inactivates and detoxifies a large panel of anticancer drugs, including sorafenib. In addition, miR-27b could enhance the sensitivity of other anticancer drugs by the same mechanism, such as doxorubicin.

MiR-216a/217 can induce resistance to sorafenib by activating the TGF-β pathways via targeting SMAD7, one of the TGF-β type 1 receptor antagonists, as well as the PI3K-Akt signaling in HCC cells (23). MiR-101 could target dual specificity phosphatase 1 (DUSP1), inhibit TGF-β activation, potentiate macrophage modulation innate immune responses, and finally augment the effect of sorafenib in HCC cells (45). HCC-associated macrophages are part of the tumor microenvironment, accelerating tumor progression through releasing growth factors. Reducing TGF-β activation could alter macrophage polarization and enhance its immune responses to sorafenib. In addition, miRNA-1274a is up-regulated after sorafenib treatment, and could significantly inhibit the expression of proteolytic and metalloproteinase 9 (ADAM9) and repress MICA shedding, leading to enhanced antitumor immunity by sensitizing NK cells to sorafenib (44).

Hypoxia has been detected in various biology processes including sorafenib acquired resistance (73). Hypoxia-inducible factors (HIFs), particularly HIF-1α and HIF-2α, are a pivotal mediators of hypoxia response (74). It is proved that the level of miR-338-3p expression is significantly decreased in HCC both *in vivo* and *in vitro*, MiR-338-3p could increase sorafenib sensitivity of HCC by down-regulating HIF-1α (41).

MiR-338-3p can regulate the efficacy of sorafenib by altering transcription of downstream drug-resistance and drug-metabolism genes, which control the clearance of exogenous drugs (41). Overexpress of miR-338-3p decreases the expression of VEGF, GLUT-1, and MDR1. The transcription product of MDR1 is p-gp, which is an energy-dependent “drug pump” and energy-dependent transporter that can accelerate the clearance of antitumor drugs and induce drug resistance. MiR-140-3p targets pregnenolone X receptor (PXR), which can transfer a variety of different compounds out of cells (75), thus increasing intracellular anti-tumor drug concentration including sorafenib by down-regulation of the expression of drug-resistance-related genes (43). Similarly, miR-379 could provoke sorafenib resistance through increasing the expression of multi drug resistant protein (MRP2), which facilitates the anti-tumor drug transport and contributes to sorafenib resistance (76).

MiR-367-3p elevates the efficacy of sorafenib in HCC by functioning as an androgen receptor (AR) enhancer, resulting in dephosphorylation and inactivation of AKT, and ERK via altering the MDM2-AR-FKBP5-PHLPP signals (46). AR plays a dual role in occurrence and development of HCC and may act as a metastatic suppressor or stimulator to promote HCC initiation.

## lncRNAs Involved in Sorafenib Resistance of HCC

lncRNAs are non-coding RNAs with more than 200 nucleotides in length, which show no protein-coding potentiality (42). lncRNAs can be further divided into five categories according to the nearby relative protein-coding genes where they locate in: (a) sense lncRNAs:overlapping a protein-coding gene; (b) antisense lncRNAs: located in the antisense orientation to a protein-coding gene; (c) bidirectional lncRNAs: generated from neighboring protein-coding genes on the opposite strand; (d) intronic lncRNAs: arised from an intron of a protein-coding gene; and (e) intergenic lncRNAs (lincRNAs): located between two protein-coding genes (77). lncRNAs may function as signals, decoys, guides or scaffolds (78). They can also serve as competing endogenous RNAs (ceRNAs) through the combination of their complementary miRNA response elements (MREs) and the primary miRNAs, exerting positive or negative effects on the processing and expression of mature mRNAs, thus indirectly involved in various progresses of physiological process (79).

Several lncRNAs have been reported to play pivotal roles in the initiation and development of HCC, such as MALAT-1, HULC, and H19. However, the function of most lncRNAs remains obscure (80–82). Previous studies of lncRNAs regulation on sorafenib resistance in HCC and the mechanisms are listed in Table 2.

**Table 2:** lncRNAs involved in sorafenib resistance in HCC.

**lncRNA**	**Effect**	**Target**	**Meachinsm**	**Signaling pathway**	**References**
linc-VLDLR	Decrease	—	Facilitate drug metabolism	—	(83)
THOR	Decrease	—	Promote CSC formation	TGF-β	(84)
linc-ROR	Decrease	—	Promote CSC formation	TGF-β	(85)
lncRNA SNHG3	Decrease	MiR181	Promote EMT	PI3K/AKT	(86)
lncRNA TUC338	Decrease	RASAL1	—	MAPK	(87)

*“—“: unknown*.

*linc-VLDLR, Involvement of extracellular vesicle long non-coding RNA; THOR, testis-associated highly conserved oncogenic long non-coding RNA; linc-ROR, regulator of reprogramming; SNHG3, small nucleolar RNA host gene 3; TUC338, transcribed ultra-conserved region 338; RASAL1,The RAS GTPase-activating-like protein 1*.

### lncRNAs and Drug Efflux System

linc-VLDLR is a large intergenic non-coding RNA, which can be delivered by extracellular vesicles (EVs) and provoke sorafenib resistance of HCC (83). In detail, linc-VLDLR modulates the expression of drug transporter genes, such as ATP-binding cassette, subfamily G member 2 (ABCG2), bringing decreased sensitivity to a variety of anti-cancer drugs including sorafenib (88).

Both the content of linc-VLDLR and the secretion of EVs significantly increase after the exposure to sorafenib of HCC cells, and the content of linc-VLDLR is also increased within EVs. EVs could transmit chemoresistant elements from drug-resistant tumor cells to sensitive ones and make them less sensitive. Down-regulation of VLDLR can significantly reduce the expression of ABCG2 mRNA and protein in recipient cells owing to EVs' function.

Apart from linc-VLDLR, ROR, and TUC338 can also transmit intracellular signals through EVs, affecting the response of HCC cells to sorafenib. This has offered a new insight into sorafenib resensitization via lncRNAs delivered by EVs.

### lncRNAs and CSCs

Testis-associated highly conserved oncogenic long non-coding RNA (THOR) acts as an oncogene in HCC. A dramatic high expression of THOR is observed in both sorafenib-resistant HCC cells and live CSCs. THOR promotes liver CSC generation via β-catenin pathway, and its own expression is regulated by TGF-β/SMAD signaling pathway. Down-regulation of THOR expression could significantly inhibit the expansion of liver CSCs by inhibiting their dedifferentiation and the self-renewal abilities (84).

Additionally, lincRNA-ROR is also involved in provoking sorafenib resistance by enhancement of CSC generation via regulation of the TGF-β pathway (85). TGF-β activation reduces the sensitivity of HCC cells to sorafenib and promotes both the secretion of extracellular vesicles and linc-ROR concentration within these vesicles.

### lncRNAs and EMT

During EMT, epithelial cells lose most of their epithelial characteristics and acquire many of the characteristics of mesenchymal cells, leading to enhanced ability of cell migration, invasion, and anti-apoptosis, thus making the tumor cells more mobile and resistant to anti-tumor drugs including sorafenib (89).

A series of lncRNAs have been proved to be implicated in EMT, including HULC, H19, CASC2, TUG1, PVT1, HOXA-AS2, HOST2, CASC2, ANRIL, and so on (90–97). As in HCC, up-regulation of small nucleolar RNA host gene 3 (SNHG3) is reported to be related to sorafenib resistance by altering the miR-128/CD151 pathway. Functioning as a ceRNA, SNHG3 inhibits the biological function of miR-128 by interfering its binding to the target CD151 mRNA. MiR-128 functions as a negative regulatory in proliferation, invasion, migration, drug resistance, and apoptosis resistance in various types of cancers (98). CD151 can activate PI3K/AKT signaling to promote EMT in HCC cells (86). SNHG3 expression is remarkably increased in highly metastatic HCC cells. SNHG3 up-regulation promotes sorafenib resistance of lowly metastatic HCC cells.

### Other Mechanisms

It has been reported that lncRNAs can manipulate the efficacy of sorafenib in HCC cells by participating in various signaling pathways, including TGF-β pathway, MAPK pathway and PI3K/AKT pathway. As mentioned above, THOR and ROR induce sorafenib resistance by activating TGF-β pathway (85, 99). Recently, the transcribed ultra-conserved region 338 (TUC338) is reported to provoke sorafenib resistance of HCC cells by targeting RASAL1, a negative regulator in MAPK pathway activation by catalyzing RAS inactivation (87).

## Conclusion and Future Directions

Accumulating evidence indicates that ncRNAs could be used as novel biomarkers for sorafenib sensitivity and clinical prognosis, as well as promising therapeutic targets to re-sensitize sorafenib-resistant HCC patients. miRNAs regulate sorafenib sensitivity by binding to the 3′-UTR of targeted mRNAs. While lncRNAs mainly act as sponge for miRNAs involved in regulation of drug resistance. It brings a bright research prospect that ncRNAs and/or the corresponding inhibitors combined with sorafenib would be a better choice than sorafenib alone in the battle against HCC. For example, Tang et al. (17) synthesizes an artificial lncRNA (AlncRNA) that could target multiple sorafenib-resistance-related miRNAs simultaneously, including miR-21, miR-153, miR-216a, miR-217, and miR-494, and proves its function in resensitizing the drug-resistant HCC cells to sorafenib again. Other in-depth research on ncRNA treatment methods are also under way, and some ncRNA-based therapies have entered Phase II clinical trials (100).

Notably, ncRNA-based therapies are still facing challenges in clinical practice. First, considering the poor biological stability, ncRNAs are easy to degrade in the lysosome and lose their efficacy. The problem may be overcome by constructing a more reliable delivery systems such as sorafenib-sensitive-ncRNA-loaded nanoparticles or the complementary sequence to the sorafenib-resistant-related ncRNA-loaded nanoparticles. For instance, Meng et al. constructs the P-glycoprotein (Pgp) siRNA-loaded nanoparticles and reverses doxorubicin resistance in breast cancer, providing a theoretical basis for ncRNA-based therapies in overcoming sorafenib resistance (101). Second, the off-target effect of ncRNAs may cause adverse reactions by targeting genes in normal cells, yet the preventive and handling methods have not been fully characterized. Third, the precise and quantitative application of ncRNAs is hard to grasp in clinic. Last but not least, there is still much to learn about whether HCC may be resistant to ncRNA-based therapies.

Taken together, ncRNA-based therapies in estimating and overcoming sorafenib resistance in HCC have promising prospect. Nevertheless, evidence for actual application of ncRNAs remains insufficient, asking for more clinical trials in the future.

## Author Contributions

YL and BF were the major contributors in writing the manuscript. MA and YZ designed figures. HZ and TW performed the literature search. XC and PC revised the manuscript. RW gave a main concept, supervised work, and he is a grant recipient. All authors took part in preparation and modification of figures and manuscript.

### Conflict of Interest

The authors declare that the research was conducted in the absence of any commercial or financial relationships that could be construed as a potential conflict of interest.
