# Corrigendum: Non-coding RNAs: Emerging Regulators of Sorafenib Resistance in Hepatocellular Carcinoma

**DOI:** 10.3389/fonc.2020.00277

**Published:** 2020-03-03

**Authors:** Yongting Lai, Bing Feng, Mubalake Abudoureyimu, Yingru Zhi, Hao Zhou, Ting Wang, Xiaoyuan Chu, Ping Chen, Rui Wang

**Affiliations:** ^1^Department of Medical Oncology, Nanjing School of Clinical Medicine, Jinling Hospital, Southern Medical University, Nanjing, China; ^2^Department of Medical Oncology, School of Medicine, Jinling Hospital, Nanjing University, Nanjing, China; ^3^Department of Medical Oncology, Jinling Hospital, Nanjing Medical University, Nanjing, China; ^4^Department of Oncology, First People's Hospital of Yancheng, Fourth Affiliated Hospital of Nantong University, Yancheng, China

**Keywords:** hepatocellular carcinoma (HCC), sorafenib resistance, non-coding RNAs (ncRNAs), microRNAs (miRNAs), long non-coding RNAs (lncRNAs)

There was an error in the Funding statement. The first funding number should be **81772995**.

The authors apologize for this error and state that this does not change the scientific conclusions of the article in any way. The original article has been updated.

